# Infantile Hypotonia: A Case of Spinal Muscular Atrophy With Respiratory Distress Type 1 Presenting As Infant Botulism

**DOI:** 10.7759/cureus.19006

**Published:** 2021-10-24

**Authors:** Juan Cardenas, Jose Cardenas, Andrew Lee, Martha Brown, Fernando Galan, Jason Scimeme, Anatalia Labilloy

**Affiliations:** 1 Pediatric Medicine, University of Florida College of Medicine – Jacksonville, Jacksonville, USA; 2 Pediatric Critical Care, University of Florida Health, Gainesville, USA; 3 Pediatric Medicine, University of Florida Health Jacksonville, Jacksonville, USA; 4 Genetics, University of Florida Health Jacksonville, Jacksonville, USA; 5 Pediatric Neurology, Nemours Children's Health System, Jacksonville, USA; 6 Pediatric Critical Care, University of Florida Health Jacksonville, Jacksonville, USA

**Keywords:** spinal muscular atrophy (sma), hypoalgesia, hyperhidrosis, arrhythmia, incontinence, areflexia, hyporeflexia, autonomic dysfunction, respiratory failure, hypotonia

## Abstract

Spinal muscular atrophy with respiratory distress type 1 (SMARD 1) is a rare autosomal recessive disease characterized by distal muscular atrophy and respiratory distress. It presents between six weeks and six months of age, with an eventual requirement of respiratory support. To date, no curative treatment to attenuate or stop the clinical deterioration has been found; therefore, supportive treatment is the corner stone of management.

We report a 12-week-old infant with SMARD1 initially diagnosed and managed as a case of infant botulism secondary to a history of significant exposure to honey. SMARD1 and infant botulism all share characteristic clinical features, namely, respiratory distress, hypotonia, and autonomic dysfunction with typical onset of less than one year of age.

This case report illustrates that SMARD1, SMA Type 1, and infant botulism share common clinical features. It is important to maintain a broad differential when evaluating an infant with hypotonia, especially when there is a lack of clinical response to conventional medical interventions directed toward the working diagnosis.

## Introduction

Spinal muscular atrophy with respiratory distress type 1 (SMARD1) is a rare type of distal SMA inherited in an autosomal recessive manner with an incidence of 1:100,000 newborns [1]. The condition is characterized by progressive and severe respiratory distress, hypotonia, and autonomic dysfunction. Symptoms typically begin between six weeks and six months of age [1].

SMARD 1 can mimic SMA Type 1 and infant botulism in presentation. We report a case of SMARD1 that presented with features akin to SMA Type 1 and infant botulism. Botulism was initially suspected due to a history of exposure to honey, a known risk factor for infant botulism, making the diagnosis of SMARD1 more confounding and less overt.

## Case presentation

A 12-week-old infant male presented to the clinic with dyspnea and grunting. He was born full-term and small for gestational age (SGA), with a weight of 2381 g. Maternal history was noncontributory. The infant was generally well for the first few months of life, although parents reported a history of intermittent constipation. Interestingly, the father was a beekeeper and the family used honey at home instead of sugar.

**Figure 1:**
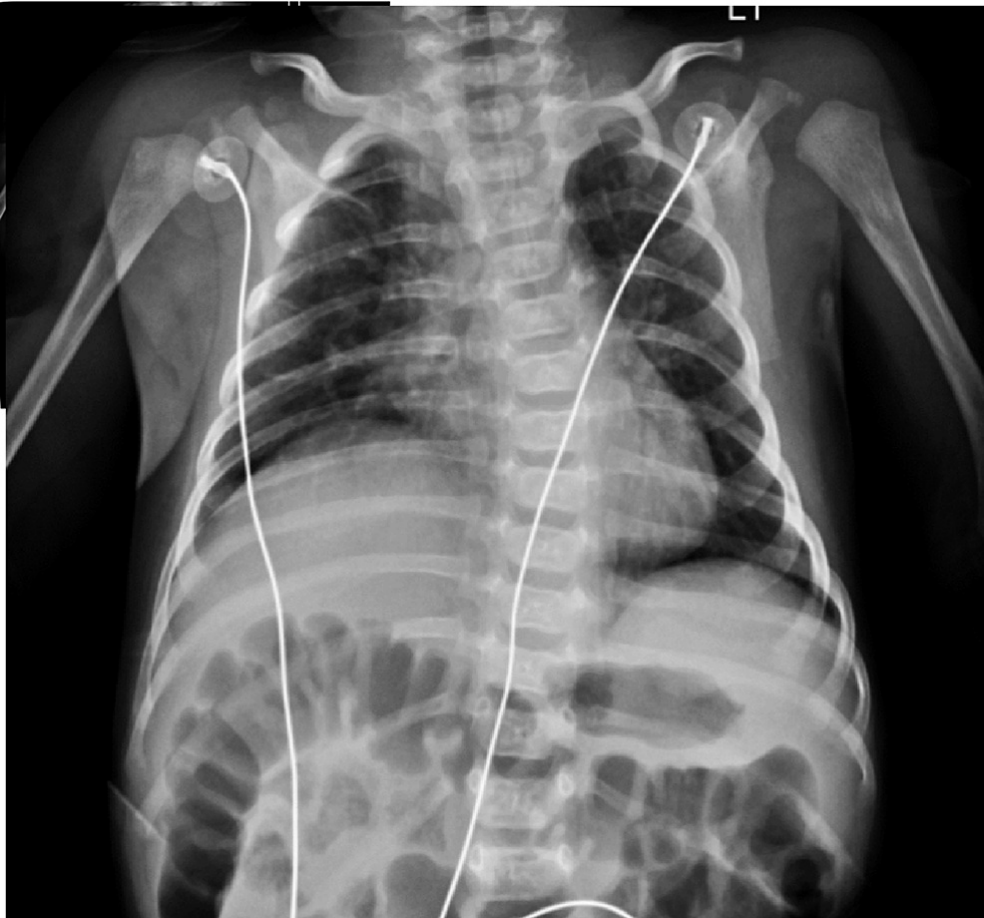
AP view of chest showing an elevated right hemidiaphragm in this infant with respiratory distress. Bilateral lung fields are otherwise clear.

The infant was sent from the clinic to the emergency department, where he was found to be severely malnourished, hypoxemic, and hypotonic. Additionally, he had a weak cry and areflexia of the lower limbs. He was consequently put on a high-flow nasal cannula (HFNC). An anterior-posterior (AP) view chest x-ray showed an elevated right hemidiaphragm (Figure 1). A head ultrasound and a limited swallow study were normal. Other tests included upper respiratory viral polymerase chain reaction (PCR) panel, severe acute respiratory syndrome coronavirus (SARS-CoV-2) PCR, and blood culture - all of which were negative. The newborn screen was normal.

**Figure 2:**
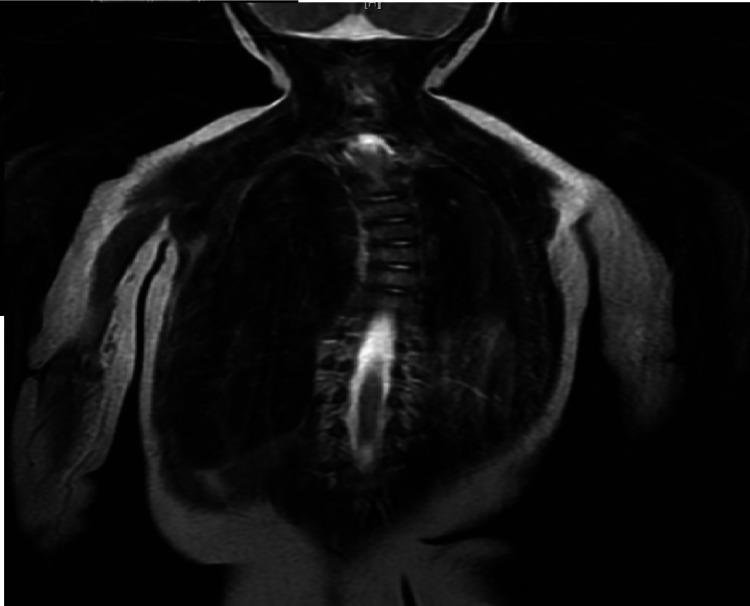
MRI showing levocurvature and dextrocurvature of the thoracic and lumbar spine, respectively. Note incidental finding of hydromyelia extending from T10-11 to T12-L1.

The infant was admitted to the pediatric intensive care unit (PICU) and was placed on bilevel positive airway pressure (BIPAP). The Infant Botulism Treatment and Prevention Program was contacted due to the significant history of honey exposure. Recommendations were to administer the Botulism Immunoglobulin Intravenous (BIGIV) and to send a stool sample to the Center for Disease Control and Prevention. During this time, Neurology and Genetics were also consulted. Neurology recommended magnetic resonance imaging (MRI) of the brain and spine which was normal, with the exception of levocurvature and dextrocurvature of the thoracic and lumbar areas, respectively (Figure 2). Genetics recommended an extensive metabolic and genetic workup, which included plasma amino acids, urine organic acids, lactate, pyruvate, acylcarnitine profile, carnitine profile, serum creatinine kinase (CK) level, Prader Willi methylation studies and SMA testing (Table 1).

**Table 1:** Metabolic and genetic workup obtained for this patient. AST: Aspartate aminotransferase; ALT: Alanine aminotransferase; CK: Creatinine kinase; T4: Thyroxine; TSH: Thyroid-stimulating hormone; IU/L: International units per liter; g/dL: Grams per deciliter; mg/dL: Milligrams per deciliter; µIU/mL: Micro international units per milliliter; ng/dL: nanograms per deciliter.

Diagnostic studies	Result	Normal value
Prader Willi/Angelman methylation studies	Negative	Negative
Whole Exome Sequencing	mutation in the immunoglobulin mu DNA binding protein 2 (IGHMBP2) gene	Negative
SMA 5q13	Negative	Negative
Urine organic acids	No specific metabolic defect	No specific metabolic defect
Pyruvic acid	0.87 mg/dL	0.30 – 1.50 mg/dL
Acyl carnitine profile	Several long and very-long-chain acylcarnitine species increased.	Low concentration
Alpha-Amino Butyric acid	Not quantitated	Not quantitated
Plasma amino acids	No specific findings	No specific findings
AST	56 IU/L	10 – 60 IU/L
ALT	67 IU/L	17 – 63 IU/L
CK	604 IU/L	49 – 397 IU/L
Albumin	3.7 g/dL	2.7 – 4.7 g/dL
Calcium	9.2 mg/dL	8.5 – 11.2 mg/dL
T4	1.43 ng/dL	1.04 – 2.86 ng/dL
TSH	4.29 µIU/mL	0.58 – 5.57 µIU/mL

After receiving BIGIV, the infant’s lower limbs were felt to be more reactive to stimuli. Deep tendon reflexes (DTRs) were improved. The infant was switched from BIPAP to HFNC and was transferred to the pediatric floor. However, this clinical improvement was short-lived. On his fourth day on the pediatric floor, the infant became hypotensive and dyspneic. He was transferred back to the PICU where he was placed on BIPAP. He became bradycardic, requiring cardiopulmonary resuscitation (CPR). The infant was subsequently intubated and received an epinephrine dose before recovering.

He remained intubated for the following three weeks. During this time, all the initial metabolic and genetic workups resulted negative. Stool botulism testing resulted in negative. Genetics recommended whole exome sequencing testing, which revealed biallelic mutations in the immunoglobulin mu deoxyribonucleic acid-binding protein 2 (IGHMBP2) gene - the gene responsible for SMARD 1.

The parents were counseled about the diagnosis, natural history and expected poor outcomes. A tracheostomy and a gastrostomy tube were placed. Palliative care was consulted, and the infant was eventually discharged home with palliative care closely followed.

## Discussion

SMARD1 is an autosomal recessive disorder caused by mutations in the IGHMBP2 gene, located on chromosome 11q13.2- q13.4.2^ ^[2]. The disease involves the destruction of the α-motor neurons in the ventral horns of the spinal cord and, as a result, manifests as distal muscular weakness, hyporeflexia and diaphragmatic paralysis [3]. Biallelic loss-of-function mutations in IGHMBP2 cause a disease spectrum that ranges from neonatal onset of severe distal motor neuropathy with diaphragmatic weakness (SMARD1) to a later onset form presenting with milder distal neuropathy only. The underlying mechanism of the abnormal IGHMP2 protein and the consequent death of the α-motor neurons of the spinal cord is at present unknown.

SMARD1 is characterized by respiratory and neurologic deterioration. Respiratory symptoms often precede motor symptoms and occur between six weeks and six months of age [1]. Respiratory failure is primarily due to diaphragmatic paralysis, which is followed by hypotonia initially involving the lower extremities. A transient or sustained improvement in muscular strength can be observed for a brief period after the initial diagnosis. Most affected individuals however have permanent hypotonia and/or paralysis of all extremities. In our case, a transient improvement of neurological reflexes and muscle strength was seen and coincided with the administration of BIGIV, which clouded the clinical picture, leading to a presumed diagnosis of botulism. However, given that the improvement was not sustained, the workup was broadened to whole-exome sequencing, which revealed the biallelic pathogenic variants in IGHMBP2.

The leading cause of death in patients with SMARD1 is respiratory failure with other common symptoms consistent with autonomic dysfunction including areflexia, bladder and bowel incontinence, constipation, arrhythmia, hyperhidrosis, hypoalgesia, fasciculations and scoliosis usually of the thoracic and lumbar spine [3,4]. Foot deformities and the development of fat pads are also common and result from adipose tissue accumulating in the phalanges following the muscular degeneration of the feet and hands [3].

The infant in our case was born SGA and had a history of constipation prior to respiratory distress and subsequent hypotonia. SMA 5q13, which is tested in our state newborn screen came back negative. The chest x-ray was remarkable for the elevated right hemidiaphragm which connotes paralysis of the phrenic nerve. Of note, unilateral paralysis, typically the right, is more commonly seen in SMARD1 than bilateral involvement [1].^ ^Our infant also had scoliosis of the spine - a frequent finding in SMARD1.

SMARD1 can be suspected when an infant, usually born less than the third percentile for weight, suddenly presents with respiratory distress that is associated with diaphragmatic paralysis [1]. Diaphragmatic paralysis is suggestive of SMARD1, especially when one or more of the following are present: family history of sudden infant death syndrome; consanguinity; weakness of the lower limbs; and/or distal skeletal or joint abnormalities^ ^[5]. Diagnosis is confirmed by gene sequencing which confirms the presence of the biallelic mutations in the IGHMBP2 gene. Other ancillary modalities to aid in diagnosis include electromyography, motor nerve conduction studies and an absent motor response after maximal stimulation.

To this date, there is no curative treatment for SMARD1 [1,6]. Management is primarily supportive. Most individuals succumb to respiratory failure before 13 months of age. Still, the natural history and long-term clinical outcomes of affected infants remain incompletely described. Potential pharmacological strategies being studied at present include cell therapy and gene therapy with IGHMBP2 gene transfer and gene correction with clustered regularly interspaced short palindromic repeats (CRISPR/Cas9) [7].

In this case, the differential diagnosis included SMA Type 1 and infant botulism. Infant botulism was initially suspected due to potential exposure to honey - a significant risk factor for the disease, along with a normal newborn screen, which ruled out SMA Type 1. In the United States (US), there is an average of seven cases of infant botulism reported annually [8]. The reported incidence of SMA is 1:6,000 to 1:10,000 live births, with approximately 50% of the cases of SMA Type 1. With regard to SMARD1, the estimated incidence is approximately one in 100,000 live births [1].

SMA is a spectrum of autosomal recessive diseases characterized by degeneration of the alpha neurons in the anterior horn of the spinal cord [9]. The most common cause of SMA is a mutation in the survival motor neuron 1 (SMN 1) gene located in chromosome 5q13 which can be divided into subtypes based on the onset and severity of the disease (Table 2) [10]. There are other types of SMA that are caused by mutations not related to the SMN 1 gene such as SMARD 1 [11].^ ^These non-5q13-related subtypes are not detected by the newborn screen. SMA Type 1 is the subtype that most resembles SMARD 1; affected individuals usually present by six months of age with severe hypotonia, dysphagia, tongue fasciculations, poor suck and breathing difficulties.

**Table 2:** Types of 5q13 SMA

Type	Onset	Life Expectancy
0	Prenatal	Fatal at birth without support
I	Less than 6 months	Less than 2 years
II	6 to 8 months	10 – 40 years
III	More than 18 months	Indefinite
IV	More than 5 years	Indefinite

Infant botulism may mimic the clinical scenario. It is caused by toxins produced by the bacteria, Clostridium difficile, which binds to the cholinergic receptors in the presynaptic cell membrane of the voluntary motor and autonomic neuromuscular junctions. Affected children are typically less than one year of age and have profound hypotonia, cranial nerve palsy, flaccid paralysis and diaphragmatic weakness that leads to respiratory failure [8].

SMARD1, SMA1 and botulism compared

The hallmarks of SMARD1 are respiratory distress, neuromuscular involvement and autonomic dysfunction. This presentation is not pathognomonic of SMARD1 and these findings can overlap with other conditions, including SMA Type 1, which has a higher population prevalence. In contrast to SMARD1, the hypotonia of SMA Type 1 affects the proximal muscles, compared to the distal involvement of SMARD1, and has less frequent respiratory symptoms [3,9].

In comparison to SMARD1, infantile botulism typically affects infants less than one year of age. Affected infants have constipation, poor feeding, hypotonia, and respiratory failure [8]. The possible exposure to Clostridium botulinum spores in raw honey products is pivotal in the diagnosis, which in this case, acted as a significant confounder in the diagnosis of SMARD1. The identification of these spores, and less frequently the toxin, is diagnostic of the infection [8].

Other neuromuscular disorders presenting as infantile hypotonia or as limb-girdle weakness should also be considered in the differential diagnosis (Tables 3, 4).

**Table 3:** Differential diagnosis of neuromuscular disorders

Subgroups of Neuromuscular disorders	Diseases
Anterior horn cell disorders	Acute infantile spinal muscular atrophy, Traumatic myelopathy, Hypoxic ischemic myelopathy, Arthrogryposis multiplex congenita
Congenital motor or sensory neuropathies	Charcot-Marie Tooth disease, Congenital hypomyelinating neuropathy, Dejerine-Sottas disease, Hereditary and autonomic neuropathy
Neuromuscular junction disorders	Transient acquired neonatal myasthenia, congenital myasthenia, magnesium toxicity, aminoglycoside toxicity, infantile botulism
Congenital myopathies	Nemaline myopathy, central core disease, multiminicore disease, Centronuclear myopathies, congenital fiber type disproportion myopathy
Muscular dystrophies	Duchenne and Becker muscular dystrophy, Classic form of congenital muscular dystrophy, Walker-Warburg disease, muscle-eye-brain disease, Fukuyama disease, Congenital muscular dystrophy with cerebellar atrophy/hypoplasia, Congenital muscular dystrophy with occipital agyria, Early infantile facioscapulohumeral dystrophy, congenital myotonic dystrophy, others.

**Table 4:** Differential diagnosis of hypotonia

Groups	Diseases
Chromosomal disorder	Down syndrome, Prader Willi, Trisomy 18, Trisomy 13, microdeletions and microduplication syndromes.
Metabolic	Disorders of glycosylation, disorders of creatine metabolism, carnitine cycle defects, fatty acid oxidation defects, glycogen storage diseases, disorders of glycogen metabolism, mitochondrial myopathies, organic acidemias, peroxisomal disorders, urea cycle defects, others.
Endocrine	Hypothyroidism, Hypoglycemia, others.
Infectious	Sepsis, meningitis, encephalitis
Neuromuscular disorders	Anterior horn cell disorders, Congenital motor or sensory neuropathies, Neuromuscular junction disorders, congenital myopathies, Muscular dystrophies, others.

In young infants who present with respiratory failure, constipation, hypotonia and poor feeding, it is important to rapidly identify the accurate diagnosis to facilitate the initiation of appropriate treatment, stop the diagnostic odyssey for alternative diagnoses and provide information to the parents for genetic counseling. While genetic testing to confirm the diagnosis of SMARD 1 was important in this case, it should not prevent consultation with the infant botulism team and the initiation of treatment with BIGIV, should it be recommended by the team.

## Conclusions

SMARD1 is an extremely rare cause of respiratory distress, neuromuscular, and autonomic dysfunction among infants. Neurologic outcomes are poor and infant mortality is primarily due to respiratory failure. This clinical presentation may overlap with other neuromuscular disorders such as SMA Type 1 and infant botulism. Therefore, the differential diagnosis should include SMARD1, especially when SMA newborn screen is negative and the clinical course and response to conventional medical interventions do not support the current diagnosis. This rare diagnosis and management require a multi-disciplinary approach, with close communication between specialists and strong support for families. Whole exome sequencing has allowed for accurate diagnosis for SMARD1, yet there is a continued need for further reported cases and studies to develop effective therapeutic strategies for SMARD1.

## References

[1] Saladini M, Nizzardo M, Govoni A, Taiana M, Bresolin N, Comi GP, Corti S (2020). Spinal muscular atrophy with respiratory distress type 1: clinical phenotypes, molecular pathogenesis and therapeutic insights. J Cell Mol Med.

[2] Grohmann K, Schuelke M, Diers A (2001). Mutations in the gene encoding immunoglobulin mu-binding protein 2 cause spinal muscular atrophy with respiratory distress type 1. Nat Genet.

[3] Perego MG, Galli N, Nizzardo M (2020). Current understanding of and emerging treatment options for spinal muscular atrophy with respiratory distress type 1 (SMARD1). Cell Mol Life Sci.

[4] Eckart M, Guenther UP, Idkowiak J (2012). The natural course of infantile spinal muscular atrophy with respiratory distress type 1 (SMARD1). Pediatrics.

[5] Vanoli F, Rinchetti P, Porro F, Parente V, Corti S (2015). Clinical and molecular features and therapeutic perspectives of spinal muscular atrophy with respiratory distress type 1. J Cell Mol Med.

[6] Al-Zaidy SA, Mendell JR (2019). From clinical trials to clinical practice: practical considerations for gene replacement therapy in SMA type 1. Pediatr Neurol.

[7] D'Amico A, Mercuri E, Tiziano FD, Bertini E (2011). Spinal muscular atrophy. Orphanet J Rare Dis.

[8] Rosow LK, Strober JB (2015). Infant botulism: review and clinical update. Pediatr Neurol.

[9] Peeters K, Chamova T, Jordanova A (2014). Clinical and genetic diversity of SMN1-negative proximal spinal muscular atrophies. Brain.

[10] Russman BS (2007). Spinal muscular atrophy: clinical classification and disease heterogeneity. J Child Neurol.

[11] Sharifi Z, Taheri M, Fallah MS (2021). Comprehensive mutation analysis and report of 12 novel mutations in a cohort of patients with spinal muscular atrophy in Iran [PREPRINT]. J Mol Neurosci.

